# Delta-24-RGD Oncolytic Adenovirus Elicits Anti-Glioma Immunity in an Immunocompetent Mouse Model

**DOI:** 10.1371/journal.pone.0097407

**Published:** 2014-05-14

**Authors:** Hong Jiang, Karen Clise-Dwyer, Kathryn E. Ruisaard, Xuejun Fan, Weihua Tian, Joy Gumin, Martine L. Lamfers, Anne Kleijn, Frederick F. Lang, Wai-Kwan Alfred Yung, Luis M. Vence, Candelaria Gomez-Manzano, Juan Fueyo

**Affiliations:** 1 Brain Tumor Center, The University of Texas MD Anderson Cancer Center, Houston, Texas, United States of America; 2 Department of Stem Cell Transplantation, The University of Texas MD Anderson Cancer Center, Houston, Texas, United States of America; 3 Department of Immunology, The University of Texas MD Anderson Cancer Center, Houston, Texas, United States of America; 4 Department of Neurosurgery, Erasmus MC, Rotterdam, The Netherlands; University of Michigan School of Medicine, United States of America

## Abstract

**Background:**

Emerging evidence suggests anti-cancer immunity is involved in the therapeutic effect induced by oncolytic viruses. Here we investigate the effect of Delta-24-RGD oncolytic adenovirus on innate and adaptive anti-glioma immunity.

**Design:**

Mouse GL261-glioma model was set up in immunocompetent C57BL/6 mouse for Delta-24-RGD treatment. The changes of the immune cell populations were analyzed by immunohistochemistry and flow cytometry. The anti-glioma immunity was evaluated with functional study of the splenocytes isolated from the mice. The efficacy of the virotherapy was assessed with animal survival analysis. The direct effect of the virus on the tumor-associated antigen presentation to CD8+ T cells was analyzed with an in vitro ovalbumin (OVA) modeling system.

**Results:**

Delta-24-RGD induced cytotoxic effect in mouse glioma cells. Viral treatment in GL261-glioma bearing mice caused infiltration of innate and adaptive immune cells, instigating a Th1 immunity at the tumor site which resulted in specific anti-glioma immunity, shrunken tumor and prolonged animal survival. Importantly, viral infection and IFNγ increased the presentation of OVA antigen in OVA-expressing cells to CD8+ T-cell hybridoma B3Z cells, which is blocked by brefeldin A and proteasome inhibitors, indicating the activity is through the biosynthesis and proteasome pathway.

**Conclusions:**

Our results demonstrate that Delta-24-RGD induces anti-glioma immunity and offers the first evidence that viral infection directly enhances presentation of tumor-associated antigens to immune cells.

## Introduction

Oncolytic viruses selectively infect and/or replicate in cancer cells, resulting in disruption of cancerous tissues while sparing normal ones [1]. These viruses, which subvert cancer cells in a multifaceted manner, are promising to overcome the resistance encountered by conventional chemo- and radio-therapies in the patients with glioblastoma, one of the deadliest cancers with dismal prognosis [1], [2]. Numerous preclinical studies have shown the feasibility and efficacy of oncolytic virotherapy in a variety of cancers [3]. Emerging preclinical and clinical evidence also suggests, in addition to the direct lysis of cancer cells, the host immune response may be critical to the efficacy of virotherapy [4]. However, the mechanism of the immunological effect is still poorly understood, especially for oncolytic human Ad5-based vectors. One main reason is the lack of an immunocompetent and replication-competent animal model for human adenovirus. Although Syrian hamster was used for evaluating the therapeutic effect of oncolytic adenovirus for several cancers [5], it is only semi-permissive for adenoviral replication. Moreover, immunological reagents are very limited in this animal system. On the other hand, although mouse cells are generally considered more deficient for adenoviral replication, a couple of mouse tumor cells are reported to be able to partially support adenoviral replication and have been used in immunocompetent mouse to evaluate the therapeutic effect of oncolytic adenoviruses [6]. In a recently report, an oncolytic adenovirus enhanced for toll-like receptor 9 stimulation increases antitumor immune responses in an immunocompetent melanoma mouse model [7]. Furthermore, one advantage of mouse model is that more materials are available for immunological studies.

In our preclinical studies, we have demonstrated that Dlta-24-RGD, a cancer-selective oncolytic andenovirus, preferentially lyses malignant glioma and glioma stem cells [8], [9]. In the immune competent context, viral infection itself and lysis of the cancer cells by the virus releases damage-associated molecular patterns (DAMPs) that can be recognized by pattern recognition receptors (PRR) expressed by cells of the innate immune system [10], [11]. The activation of PRR induces the production of large amount of proinflammationary cytokines, such as type I IFNs and IFNγ [12], [13], resulting in a Th1 immune response. As a major cytokine in many viral infections, IFNγ upregulates the expression of MHC class I [14] and three immunoproteasome subunits β1i (LMP2), β2i (MECL-1), and β5i (LMP7), which replace their constitutive counterparts, β1, β2, and β5 [15], [16], and consequently increases the activity of the MHC I antigen presentation pathway [17]. In addition, we reported previously that Delta-24-RGD induces autophagy and consequent cell lysis [9], [18]. This type of cell death facilitates efficient antigen presentation to immune cells [19], [20]. Therefore, we speculate that, during adenoviral therapy, intratumoral injection of the virus can trigger a robust innate immune response followed by an adaptive anti-tumor immunity that mediates the regression of the tumor.

Here, we set up an immunocompetent mouse glioma model for adenoviral therapy. We examined the effect of viral injections on the immune environment at the tumor site and the anti-glioma activity of the immune cells. We observed proinflammatory immune response at the tumor site stimulated by intratumoral injections of Delta-24-RGD. Consequently, the virus elicited specific anti-tumor immunity and prolonged survival of the glioma-bearing mice. Furthermore, we also investigated the direct effect of viral infection and IFNγ resulted from virus-mediated Th1 immunity on the presentation of tumor-associated antigens (TAAs) to immune cells by the tumor cells. Using an ovalbumin (OVA) modeling system, we found viral infection and IFNγ enhanced the presentation of TAA to CD8+ T cells through the canonical endogenous pathway of MHC I presentation.

## Materials and Methods

### Cell lines and culture conditions

Human glioblastoma-astrocytoma U-87 MG and lung carcinoma A549cells (American Type Culture Collection, Manassas, VA), mouse glioma GL261 cells (NCI-Frederick Cancer Research Tumor Repository, Frederick, MD) were cultured in Dulbecco's modified Eagle's medium-nutrient mixture F12 (DMEM/F12) supplemented with 10% fetal bovine serum (FBS; HyClone Laboratories, Inc., Logan, UT), 100 µg/ml penicillin, and 100 µg/ml streptomycin (Invitrogen, Carlsbad, CA). Mouse melanoma cell line B16/F10 (American Type Culture Collection), B16-OVA (a kind gift from Dr. Hong Qin, The university of MD Anderson Cancer Center, Houston, TX) [21], [22], CD8 T cell hybridoma cell line b3Z (a kind grift from Dr. Nilabh Shastri, University California, Berkeley, CA) [23] were grown in RPMI 1640 medium supplemented with 10% FBS and antibiotics. Wild-type (wt) mouse embryo fibroblasts (wtMEFs) [18] were maintained in DMEM supplemented with 5% FBS and antibiotics. The cells were kept at 37°C in a humidified atmosphere containing 5% CO_2_.

### Reagents and antibodies

IFNγ was purchased from ProSpec (East Brunswick, NJ). Ethidium homodimer 1, MG132 and Lactacystin were obtained from Sigma-Aldrich (St. Louis, MO). Brefeldin A was purchased from eBiosceince (San Diego, CA). Antibodies used in the studies were as follows: goat anti-actin (I-19) and rabbit anti-T-bet(H-210) from Santa Cruz Biotechnology (Santa Cruz, CA); rabbit anti-LC3B (where LC3B is an isoform of the autophagy marker protein light chain 3) from Cell Signaling Technology (Danvers, MA); rat anti-Mouse CD45 APC-eFluor 780, rat anti-Mouse CD4 eFluor 450, rat Anti-Mouse CD8a PerCP-Cyanine5.5, rat Anti-Mouse CD11b APC, American hamster Anti-Mouse CD11c Alexa Fluor 488, American hamster anti-Mouse CD3e FITC, mouse anti-Mouse NK1.1 PE-Cyanine7, rat Anti-Mouse Ly-6G (Gr-1) eFluor 450, mouse anti-mouse MHC I (H-2Kd) APC and mouse IgG2a APC from eBioscience; Rabbit anti-CD3 [SP7] antibody from abcam (Cambridge, MA).

### Adenovirus

The replication-competent adenovirus Delta-24-RGD [8] was amplified in A549 cells, purified by the Adenopure kit (Puresyn, Inc.,Malvern, PA), and stored at −80°C. The viral titer was assayed with the Adeno-X-Rapid Titer Kit (Clontech, Mountain View, CA) and determined as PFU/ml. The virus particle/PFU ratio was less than 50.

### Immunoblotting

The cells were collected and resuspended in PBS plus protease inhibitor cocktail (Sigma-Aldrich) and then subjected to lysis by adding an equal volume of 2× sodium dodecyl sulfate loading buffer. Then the lysates were heated at 95°C for 10 min. Equal amounts of proteins from the lysates were separated by sodium dodecyl sulfate-polyacrylamide gel electrophoresis, transferred to a nitrocellulose membrane, and probed with antibodies. Finally, the protein bands were visualized using an ECL Western blot detection system (Amersham Pharmacia Biotech, Piscataway, NJ).

### Flow cytometry

For quantification of cell lysis, cells (2–5×10^5^ cells) were stained with 8 µM ethidium homodimer 1 in phosphate-buffered saline (PBS) solution for 15 min at room temperature. For analysis of cell surface protein expression, cells (2–5×10^5^ cells) were first incubated in 100 µl primary antibody solution diluted in PBS plus 3% BSA and 1 mM EDTA. After incubation at 4°C in the dark for 30 min, the cells were washed once with 1 ml cold PBS. If second antibody was applicable, incubation procedure was repeated with secondary antibody. After washed once with PBS, the cells were finally resuspended in 0.5 ml PBS. Stained cells were then analyzed by flow cytometry using a FACScan cytometer and CellQuest software (Becton Dickinson). Fluorescence emissions from 10^4^ cells were analyzed.

### Virus replication assay

Cells were seeded at 5×10^4^ cells/well in 12-well plates and infected with Delta-24-RGD at 10 PFU/cell. Forty-eight hours after infection, the titers of the infectious viral progeny were determined using the Adeno-X-Rapid Titer Kit (Clontech) according to the manufacturer's instruction. Final viral titers were determined as PFU/ml.

### Animal studies

GL261 cells (5×10^4^) were grafted into the caudate nucleus of the C57BL/6 mice using a guide-screw system as previously described [8]. After tumor cell implantation, mice were intratumorally injected with 5 µl of Delta-24-RGD (3×10^8^ pfu) or PBS. All mouse experimental protocols were approved by the Office of Research Administration, Institutional Animal Care and Use Committee (IACUC), The University of Texas M. D. Anderson Cancer Center and followed National Institutes of Health and United States Department of Agriculture guidelines. The animals were monitored on daily basis and were euthanized when they demonstrate moribund behavior including: slight head tilt, hemiparesis, hunched posture, scleral edema, inability to access food/water, weight loss >20% of baseline, and excessive tumor burden as indicated by doming of cranium >0.5 cm, or if show signs of lower extremity weakness. The animals were sacrificed with CO2 inhalation. To minimize suffering of the animals, ketamine/xylazine or buprenorphine was given for signs of pain, eye wincing, hunched state with front limbs over the head.

### Immunohistochemistry

To detect proteins in the tumor xenografts, paraffin-embedded sections of the mouse tumors were deparaffinized and rehydrated with xylene and ethanol following conventional procedures [24]. Endogenous peroxidase activity was quenched by incubating the sections in 0.3% hydrogen peroxide in methanol for 30 minutes. The sections were then treated with primary antibodies at 4°C overnight. For immunohistochemical staining, Vectastain ABC kits from Vector Laboratories (Burlingame, CA) were used according to the manufacturer's instructions.

### Preparation of cell suspension of mouse brains

Mouse hemispheres with tumor were collected and placed in cell strainer (100 µm) in petri dishes with RPMI 1640. Then the hemisphere was smashed through the cell strainer into the dish. The mixture in the dish was gently pipetted up and down, brought up to 20 ml and filtered through 40 µm cell strainer. The cells were pelleted by centrifugation at 350 g for 7 min at room temperature and finally resuspended in PBS plus 1 mM EDTA.

### Preparation and stimulation of splenocytes

Mouse spleens were collected and placed in cell strainer (100 µm) in petri dishes with RPMI 1640, then were smashed through the cell strainer into the dish. The mixture in the dish was gently pipetted up and down, brought up to 10 ml. The cells were pelleted by centrifugation at 350 g for 7 min at room temperature and resuspended in Red Blood Cell Lysing Buffer Hybri-Max (Sigma-Aldrich) to lyse red blood cells according to the manufacturer's instruction. To activate the splenocytes, 5×10^4^ target cells pre-fixed with 1% paraformaldehyde (PFA) were incubated with 1×10^6^ splenocytes per well of a round-bottom 96-well plate for 40 hours. The concentration of IFNγ in the supernatant was assessed with standard ELISA assay.

### Gl261-OVA cell line

First, chicken ovalbumin (OVA) cDNA (Origene, Rockville, MD) was subcloned into a lentivector plasmid pCDH-CMV-MCS-EF2-Puro (System Biosciences, Mountain View, CA). The lentivirus expressing OVA was packaged in ViralPower Lentiviral Expression Systems (Invitrogen) and concentrated with Lenti-X Concentrator (Clontech) according to the manufacturer's instruction. Then GL261 cells were infected by the virus and stable cell line was established by puromycin selection at 1 µg/ml.

### Detection of antigen presentation by LacZ T cell hybridoma assay

Antigen cross-presentation of OVA257–264 was detected using the CD8 T cell hybridoma cell line B3Z that expresses β-galactosidase under control of the IL-2 promoter [23]. Hybridoma cells (1×10^5^) were incubated with 5×10^4^ target cells pre-fixed with 1% PFA (100 µl total volume) per well of a round-bottom 96-well plate. 18 h later, the β-galactosidase activity was assessed with the Beta-Glo Assay System (Promega, Madison, WI) according to the manufacturer's instruction.

### Statistical analyses

A two-tailed Student's *t* test was used to determine the statistical significance of the results of our experiments. *P* values of <0.05 were considered statistically significant. Data are given as Mean ± standard error (S.E.). The animal survival curves were plotted according to the Kaplan–Meier method [25]. Survival in different treatment groups was compared using the log-rank test.

## Results

### Delta-24-RGD induces cytotoxicity in mouse glioma cells

**Figure 1:**
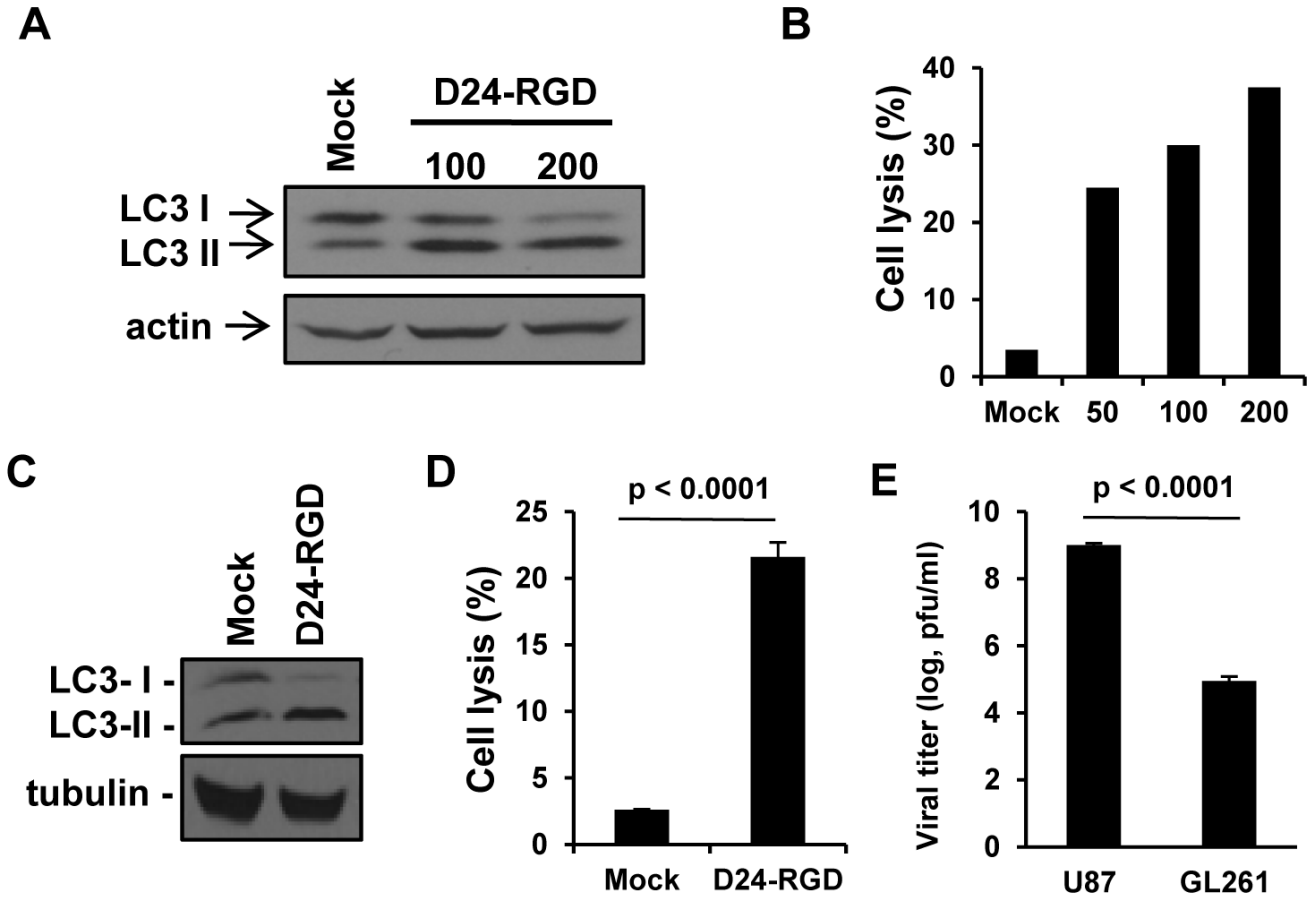
*In vitro* characterization of Delta-24-RGD-mediated autophagy and anticancer effect in GL261 mouse glioma cells. A &B. Gl261 cells were infected with Delta-24-RGD (D24-RGD) at the indicated dose (pfu/cell). 72 h later, cell lysates were analyzed by Western Blot for LC3-I to LC3-II conversion (A) and Delta-24-RGD-mediated cell lysis was quantified (B). Actin levels are shown as a protein loading control. C &D. U-87 MG cells were infected with Delta-24-RGD (D24-RGD) at 10 pfu/cell. 72 h later, cell lysates were subjected to Western Blot analysis for LC3-I to LC3-II conversion (C). Tubulin levels are shown as a protein loading control. Delta-24-RGD-mediated cell lysis was quantified (D). *P*<0.0001 (Student's t-test, double sided). (D). E, Cells were infected with Delta-24-RGD at 10 pfu/cell. 48 h later, viral progeny was quantified in the cultures. *P*<0.0001 (Student's t-test, double sided). Data are represented as mean ± S.E.; *n* = 3. Note that cell lyses and viral replication were more efficient in human than in mouse glioma cells.

In order to study oncolytic adenoviral therapy in a glioma model in immune-competent mice, we first characterized the effect of Delta-24-RGD on mouse glioma cell line GL261. As the viral infection in human cells, Delta-24-RGD started to cause the cells to round up and detached from the bottom of the culture dish 48 hours after infection at a viral dose of 100 PFU/cell. 24 hours later, autophagy was induced in these cells as indicated by the conversion of the protein LC3 I to LC3 II (figure 1A) [18]. Autophagy was associated with cell lysis which was defined by the disruption of the cell membrane resulting in the staining of the DNA in the nucleus with fluorescence dye ethidium homodimer 1 (figure 1B) [18]. Thus, Delta-24-RGD induced 25% to 40% cell death at the viral doses ranging from 50 to 200 pfu/cell. These results were in agreement with the effect of the virus in human glioma cells although at a lower dose (10 pfu/cell) the virus already displayed the same efficacy in human glioma cell line U-87 MG (figure 1C and D) [18]. Finally, we tested the replication of the virus in both human and mouse glioma cell lines. We found Delta-24-RGD replicated about 10,000 folds more efficiently in U-87 MG cells than in GL261 cells (p<0.0001) (figure 1E). In summary, although with less potency than in human cells, Delta-24-RGD induces autophagy and cell lysis in mouse glioma GL261 cells, which could contribute to the induction of immune response against the tumor in vivo [26], [27]. To compensate the deficiency of the viral replication in mouse glioma cells, we later treated the glioma xenografts with multiple intratumoral injections of the virus in in vivo studies.

### Intratumoral injection of Delta-24-RGD activates immunity at the tumor site

**Figure 2:**
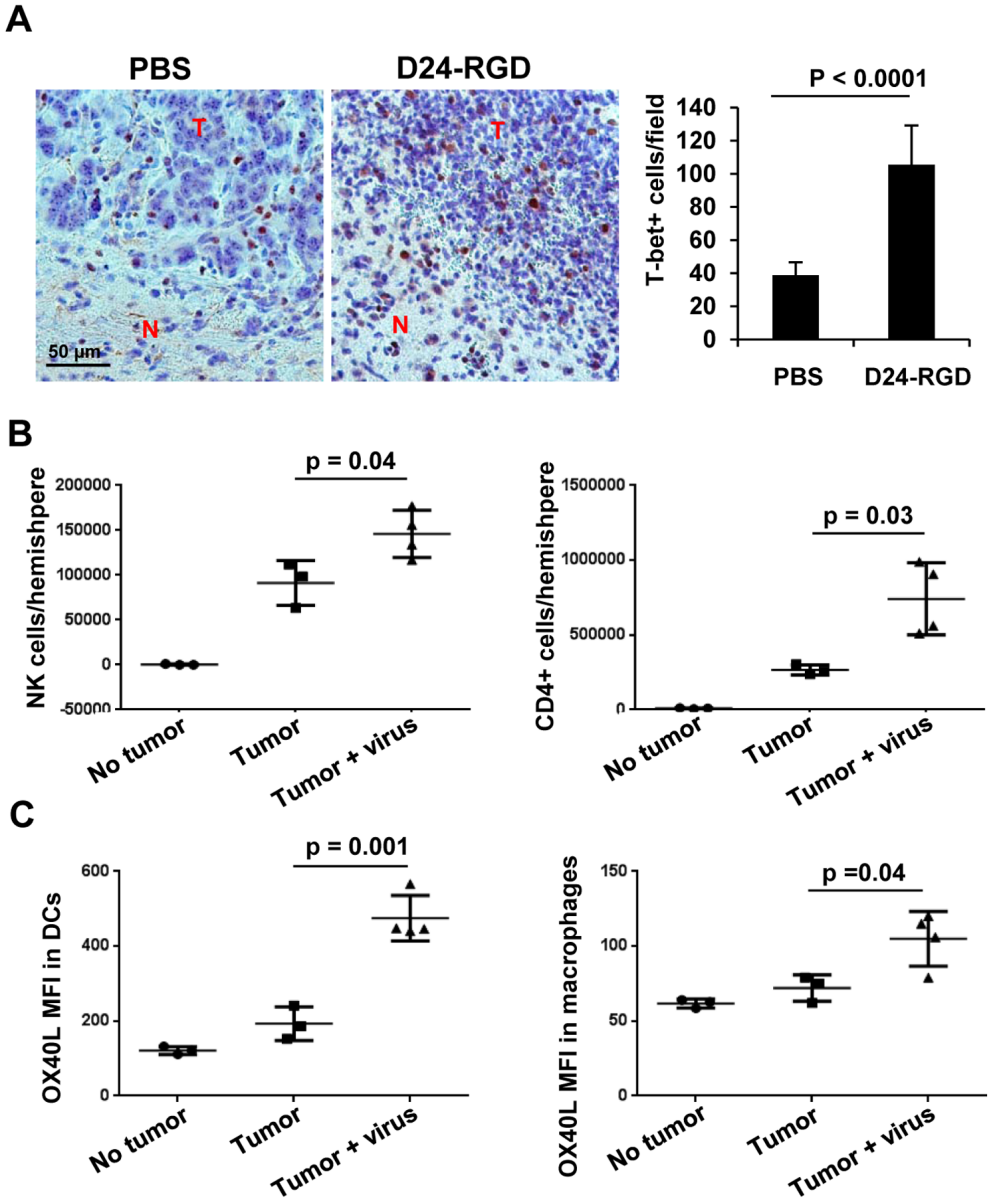
Delta-24-RGD induced immune cells infiltration in GL261 mouse glioma xenografts. Delta-24-RGD was administered intratumorally on days 7, 9, 11 after GL261 cell intracranial implantation. On day 14 of the experiment, brains were collected and analyzed. The brains were fixed (A) or the leukocytes from fresh hemispheres with or without tumor were isolated and analyzed with flow cytometry (B and C). A. Immunohistochemistry analysis of T-bet expression in paraffin-embedded tumor sections from mice treated with PBS or Delta-24-RGD (D24-RGD) (*left panel*). T-bet+ cells were quantified and represented as mean ± S.E.; *n* = 9–10. *P*<0.0001 (Student's t-test, double sided). (*right panel*). T: tumor; N: normal brain tissue. B. NK+ cells or CD4+ T cells present in hemispheres with or without tumor were quantified. No tumor: naïve mice; Tumor: GL261-glioma bearing mice; Tumor + virus: Delta-24-RGD-treated GL261-glioma bearing mice. Data are represented as mean ± S.E.; *n* = 3–4. *P*<0.05 (Student's t-test, double sided). C. OX40L expression levels in dendritic cells (DC) or macrophages obtained from brains of naïve mice (*No tumor*), GL261-glioma bearing mice (*Tumor*), and Delta-24-RGD-treated GL261-glioma bearing mice (*Tumor + virus*). MFI: mean fluorescence intensity. Data are represented as mean ± S.E.; *n* = 3–4. *P*<0.05 (Student's t-test, double sided). Note that increase in T-bet positive cells (cells expressing IFNγ), CD4, and enhanced expression of OX40L in dendritic cells and macrophages strongly indicate a Th1 immunity at the tumor site.

**Figure 3:**
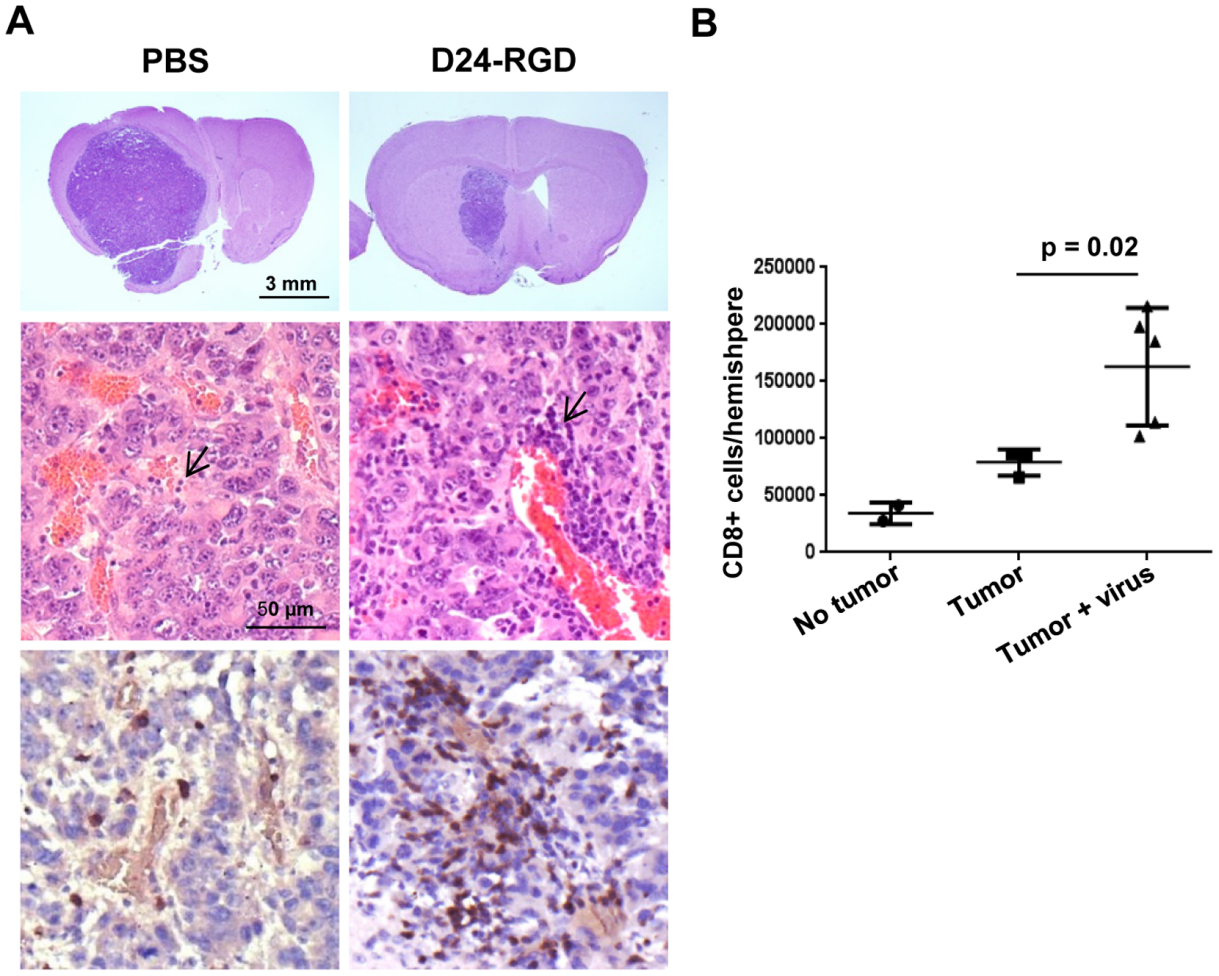
Delta-24-RGD treatment mediated tumor shrinkage and lymphocytic infiltration. 20 days after GL261 implantation and 9 days Delta-24-RGD injections, as indicated in Figure 2, the murine brains were fixed (A) or the leukocytes from fresh hemispheres with or without tumor were isolated and analyzed with flow cytometry (B). A. Brains were stained with hematoxylin-and-eosin (*upper two panels*) or immune stained for CD3 (*bottom panel*). Note that, as expected, lymphocytes as round cells with little visible cytoplasm and dense and monochromatic nucleus (arrows) are more prominent in perivascular areas. B. CD8+ T cells were quantified and data represented as mean ± S.E.; *n* = 3–4. *p* = 0.02 (Student's t-test, double sided). No tumor: naïve mice; Tumor: GL261-glioma bearing mice; Tumor + virus: mice with Delta-24-RGD-infected GL261-glioma bearing mice. Note that the viral injections resulted in accumulation of lymphocytes, and, importantly, a significantly higher number of CD8+ cells at the tumor site.

Next, we tested the effect of Delta-24-RGD on the immune environment at the tumor site in a syngeneic GL261 glioma model in C57BL/6 mice. To this end, immunohistochemistrty analysis demonstrated increased presence of T-bet expressing cells within the tumor 72 hours after three viral injections in GL261 xenografts in the brain (figure 2A). Since T-bet is a master transcription factor regulating the commitment to the T helper 1 (Th1) cell lineage [28], our data suggest that the virus stimulates Th1 type immunity within the tumor microenvironment. In addition, flow cytometry analysis also showed an increment in the populations of NK cells (CD45+ CD3 low, NK1.1+) and CD4+ lymphocytes (CD45+ CD4+) at the tumor site after viral injections in the tumor (p<0.05), indicating both innate and adaptive immunity were induced by the viral infection (figure 2B). Moreover, the expression of OX40 ligand (OX40L) on dendritic cells (DCs, CD45+ CD11b+ CD11c+) and macrophages (CD45+ CD11b+ GR-1-) increased by Delta-24-RGD injections (figure 2C). OX40L is not constitutively but can be induced on professional antigen presenting cells (APCs) such as DCc and macrophages [29]. Its receptor, OX40, is transiently expressed after T-cell receptor engagement and is upregulated on the most recently antigen-activated T cells within inflammatory lesions [29]. Since OX40/OX40L pathway enhances the activation of effector T cells [30], these results indicate that Delta-24-RGD injections in the tumor activate these two APC populations to present more efficiently viral and TAAs to T effector cells. As a consequence, nine days after three viral injections, pathological studies revealed that virus treatment resulted in tumor inhibition (figure 3A, upper panel) that was coincident with increased lymphocyte populations (figure 3A middle panel) including CD3+ cells (figure 3A lower panel) presented in the treated tumors compared to the control ones. Further, we observed increased CD8+ lymphocytes, the cytotoxic T cells to fight against the tumor cells, at the tumor site with flow cytometry analysis (p = 0.02) (figure 3B).

### Delta-24-RGD primes an anti-glioma immune response and prolongs the survival of glioma-bearing mice

**Figure 4:**
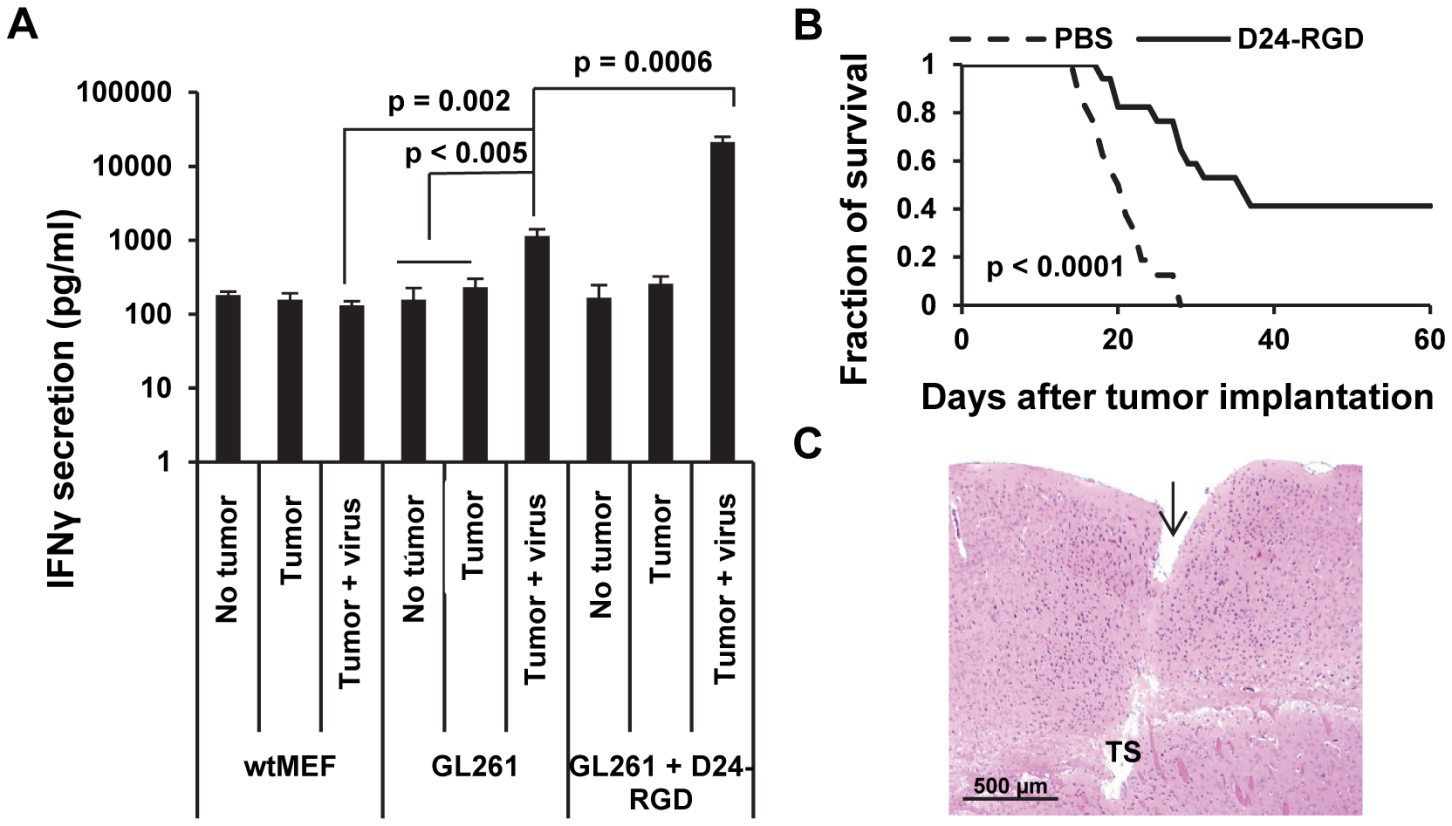
Delta-24-RGD triggered anti-tumor immunity and prolonged the survival of glioma-bearing mice. A. Delta-24-RGD was injected into the intracranial tumor on days 4, 7, 9 after tumor implantation. On day 15, the spleens of 4 mice from each group were harvested, grouped together, and the splenocytes were isolated and incubated with the indicated target cells (wt-MEFs, Gl261, or Delta-24-RGD-infected Gl261). Then, IFNγ secreted by the splenocytes was quantified by ELISA. No tumor: naïve mice; Tumor: mice with GL261-glioma; Tumor + virus: mice with GL261-glioma treated with Delta-24-RGD. Data are represented as mean ± S.E.; *n* = 3. *P*<0.05 (Student's t-test, double sided). B. Kaplan–Meier curves of overall survival of Delta-24-RGD–treated (*n* = 17) and PBS–treated (*n* = 16) glioma-bearing mice. *P*<0.0001 (Log-rank test). C. Histological examination of the brains from the long-term surviving Delta-24-RGD-treated mice. Shown is a close-up of a brain section from a representative mouse that was sacrificed 91 days after tumor implantation. The brain was fixed and stained with hematoxylin-and-eosin. Note the needle track (arrow) and tumor sequela (TS) in the implantation site.

We hypothesized that viral infection prime a tumor-specific immune response. To test this hypothesis, we isolated the splenocytes, which are a reliable window of Th1 immunity and a rich source of antigen-specific T cells, from naïve and GL261 glioma bearing-mice with or without Delta-24-RGD treatment. Then the splenocytes were incubated with PFA-fixed target cells: nonspecific control wtMEFs, GL261 and virus-infected GL261 cells. After 40 hours, IFNγ secreted by the splenocytes, which indicates the specific activity against the target cells, was assessed with ELISA. We found that only splenocytes from the virus-treated glioma-bearing mice, but not from untreated or naïve mice, reacted with GL261 glioma cells (p<0.005) (figure 4A). Further, the reaction was tumor-specific because it was not observed with wtMEFs (p = 0.002) (figure 4A). In addition, the reaction was much stronger with Delta-24-RGD-infected GL261 cells (p = 0.0006) (figure 4A), suggesting the immune response was elicited by the antigens from both the tumor cells and the virus or the viral infection enhanced presentation of TAAs to splenocytes. As a consequence, the survival of glioma-bearing mice was prolonged, from median survival of 20.5 days in control group to 36 days in virus-treated group (figure 4B) (p<0.0001). The treatment also induced about 40% long-term survival (figure 4B), indicating complete regression of the tumor in the mice. Pathological analysis of the brains from the surviving mice showed no signs of tumor (figure 4C). Different from the viral treatment of human glioma xenografts in athymic mice which are permissive for viral replication and spread [8], we didn't observe big necrotic cavities induced by the oncolysis of the tumor by the virus, suggesting the regression of the tumor is not due to direct lysis activity of the virus at the injection site.

### Delta-24-RGD infection and IFNγ enhance the presentation of TAA to CD8+ T cells

**Figure 5:**
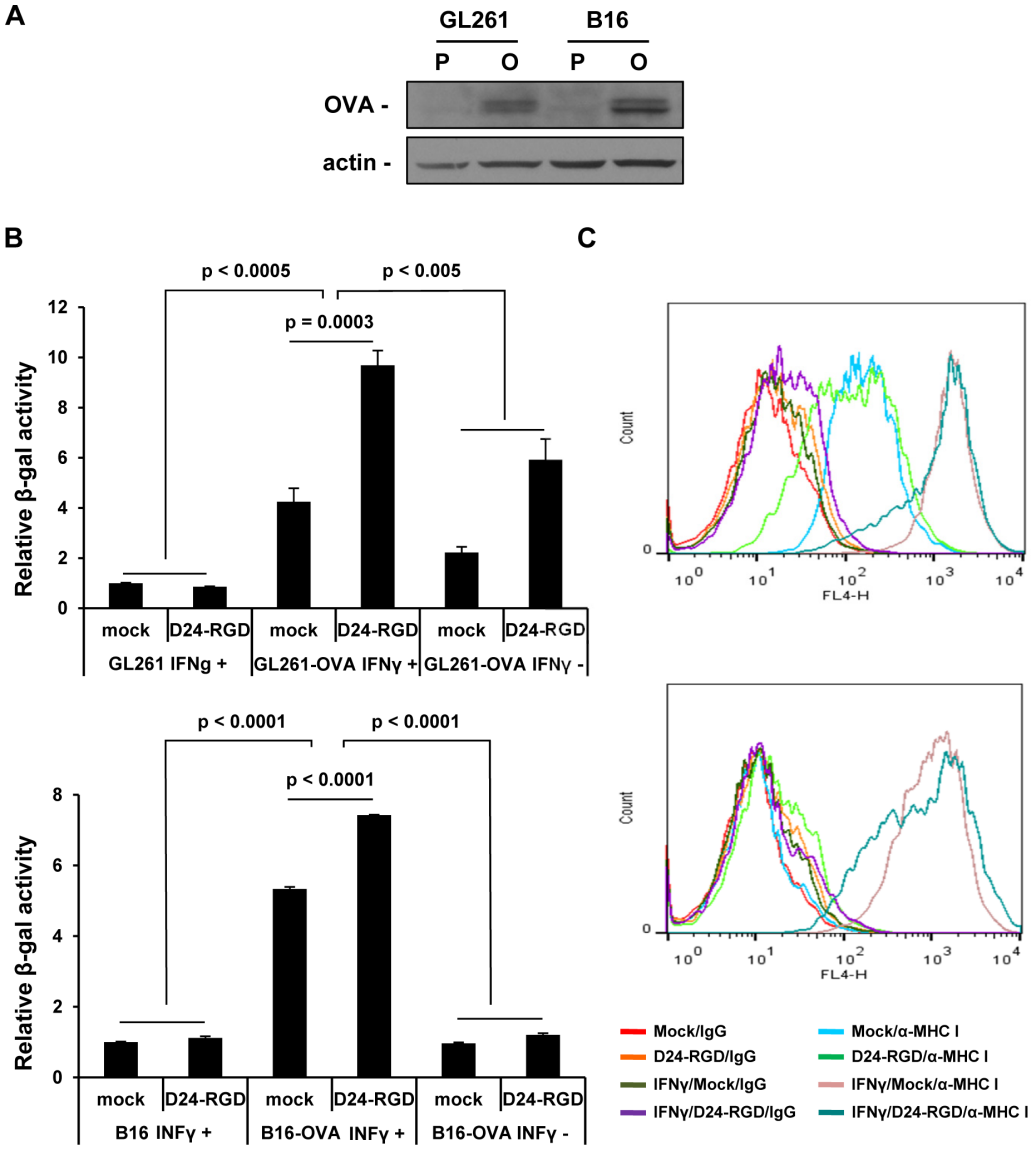
Delta-24-RGD infection and IFNγ increased the presentation of TAA to CD8+ T cells. A. OVA expression in GL261-OVA and B16-OVA cells. Whole cell lysates were analyzed by immunoblotting for the expression of OVA protein. Actin protein levels are shown as a loading control. P: parental cells; O: OVA-expressing cells. B. Presentation of OVA antigen by OVA-expressing tumor cells to OVA-specific CD8+ T cells. Cells were infected with Delta-24-RGD (GL261 and GL261-OVA at 100 pfu/cell, B16 and B16-OVA at 20 PFU/cell) in the presence or absence of IFNγ (200 u/ml, added 4hrs post infection) for 48 h. After fixed with PFA, cells were incubated with OVA-specific CD8+ T cells for 18 h and then the activation of the T cells (upregulation of IL-2 promoter activity) were assessed with β-Glo assay. The relative β-gal activity refers to the folds of activity compared to the activity from the group with mock-treated parental cells (assigned as 1). Data are represented as mean ± S.E.; *n* = 3. *P*<0.05 (Student's t-test, double sided). C. MHC I expression on the cell surface as assessed by flow cytometry analysis. Cells were infected with Delta-24-RGD as in B. The cells were then immunostained with mouse anti-mouse MHC I (H-2Kd) APC and mouse IgG2a APC and analyzed with flowcytometry for cell surface MHC I expression. Top panels in B and C: GL261-OVA; bottom panels in B and C: B16-OVA.

We have already shown Delta-24-RGD injection increases T-bet expressing cells within the tumor, which should result in the upregulation of Th1 cytokines including IFNγ. IFNγ is one of the consequences of viral infections and stimulates the expression of histocompatibility complex (MHC) proteins and immunoproteasome subunits to facilitate presentation of antigens to CD8+ T cells [14]–[17]. Thus we hypothesized that Delta-24-RGD infection enhances the presentation of TAAs to CD8+ T cells either through IFNγ stimulation or by the infection itself. To test this hypothesis, we set up a system in which we used OVA protein expressed by the tumor cells as a molecular model for TAA and an OVA-specific CD8+ T cell hybridoma cell line B3Z to examine the effect of IFNγ and viral infection on the direct presentation of TAA to T cells by the tumor cells. In this system, when MHC I present OVA epitope to B3Z cells, the engagement of T cell receptor on B3Z surface triggers a cascade of kinase activation, resulting in the transcription of cytokines [31]. Since B3Z has been engineered to express β-galactosidase under control of the IL-2 promoter [23], the increment of the activity of β-galactosidase in the T cells indicates to which extent the T cells are activated. To this end, OVA-expressing GL261-OVA and B16-OVA cells (figure 5A) were infected with Delta-24-RGD in the presence or absence of IFNγ. We found IFNγ increased presentation of OVA antigen to B3Z cells in both cell lines (figure 5B) (p<0.005). As expected, the activity was not observed in parental cell lines (p<0.0005), confirming that the stimulation is specific for OVA antigen. The expression levels of MHC I were correlated with the activation of B3Z cells (figure 5B and C). In GL261-OVA cells, in the absence of IFNγ, there was already MHC I expression on the cell surface which rendered the cells capable to stimulate B3Z cells (figure 5B and C upper panel). In the presence of IFNγ, the cells showed increased MHC I expression and stimulated B3Z cells more than in the absence of IFNγ (figure 5B and C upper panel, p<0.005). However, B16-OVA cells, without IFNγ, was unable to stimulate B3Z cells just as the parental cells due to the lack of MHC I expression while IFNγ induced MHC I expression and consequently the activation of B3Z cells (figure 5B and C lower panel, p<0.0001)). Importantly, viral infection itself also enhanced the presentation of OVA antigen to the T cells and the effect was stronger in GL261-OVA cells than in B16-OVA cells (128% vs. 39% increment) (Fig. 4D) (p<0.0005).

### Biosynthetic and proteasome activity is responsible for the presentation of TAA to CD8+ T cells stimulated by Delta-24-RGD and IFNγ

**Figure 6:**
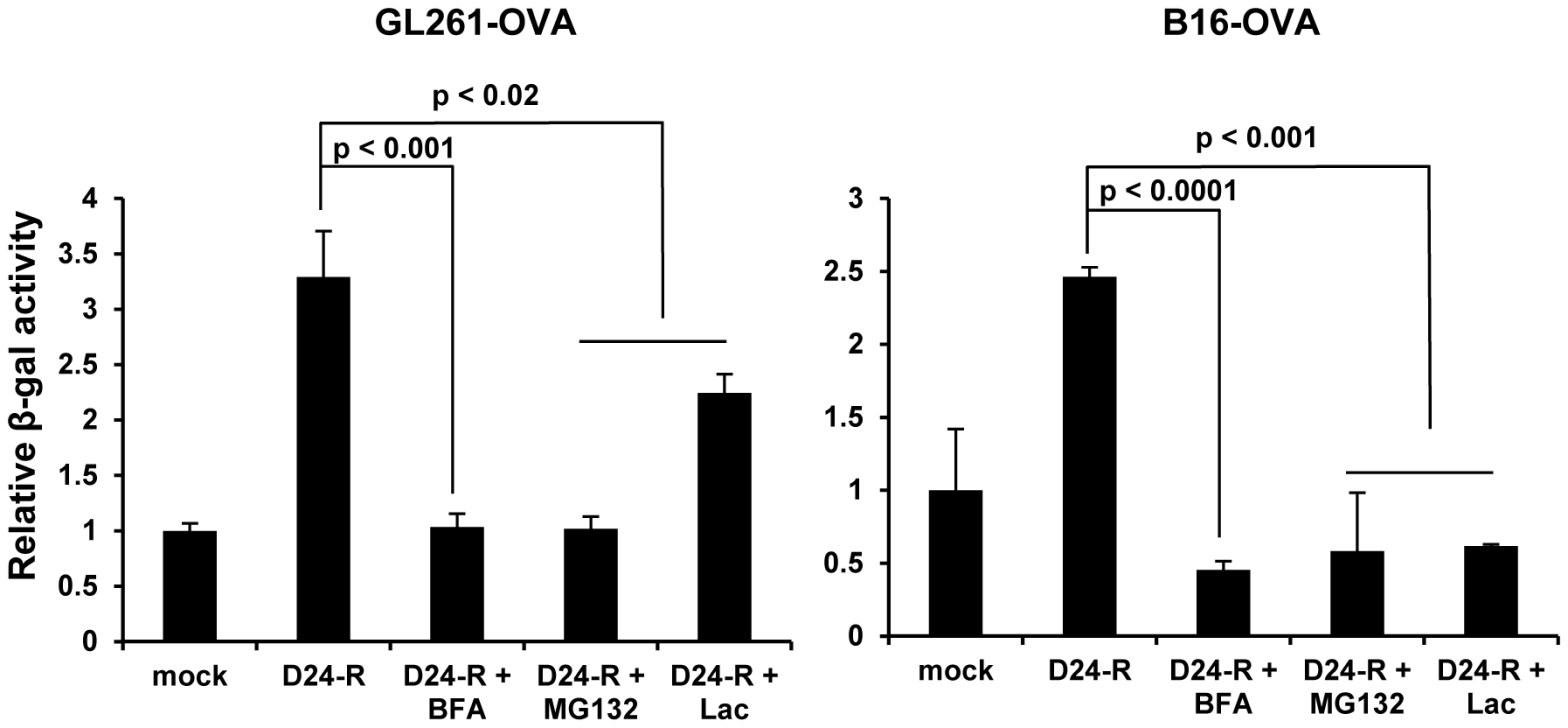
Delta-24-RGD-mediated presentation of TAA to CD8+ T cells depended on biosynthetic and proteasome activity. Cells were infected with Delta-24-RGD as in Figure 5. Brefeldin A (BFA, 6 µg/ml), MG132 (1 µM) or Lactacystin (Lac, 10 µM) was added during the last 24 h of the experiment. After fixed with PFA, the cells were incubated with CD8+ cells for 18 h and then the T-cell activation was assessed by β-Glo assay. Relative data are represented as mean ± S.E.; *n* = 3. *P*<0.05 (Student's t-test, double sided).

The presentation of antigens to CD8+ T cells is through proteasomal generation of peptides for MHC class I (MHC I)-binding to be transported to cell surface [32]. IFNγ activates both MHC I and immunoproteasome to promote this process [33]. To investigate the extent to which these activities is required for the cancer cells to present TAA to CD8+ T cells during viral infection, we treated the virus-infected cells with brefeldin A, a compound that inhibits the transport of molecules through the biosynthetic pathway [34], or proteasome inhibitors MG132 and lactacystin in the presence of IFNγ. All three drugs inhibited the presentation of OVA antigen in both GL261-OVA and B16-OVA cell lines (figure 6). These results demonstrated that processing and presentation of TAA in infected cells depend on the canonical endogenous pathway of MHC I presentation [17].

## Discussion

Emerging evidences suggest that, during virotherapy, the host immunity is an indispensable factor. It acts as a double-edged sword in regards to the efficacy and safety of the virus. Clinical experience suggests oncolytic viruses induce better response in immunosuppressed patients but associated with unacceptable toxicity [35]. However, clinical benefit is also achieved when combining virus-mediated tumor destruction with immunomodulators to attract immune recognition of TAAs [3]. Thus, although the immunity impairs the viral potency at the beginning of the infection, it is necessary for cleaning up the virus later, and importantly for the instigation of long-lasting anti-tumor immune response. In the case of glioma patients with adenoviral therapy, we speculate, at the beginning of intratumoral viral injection, the immunosuppressive tumor microenvironment gives a window for efficient viral replication. Then the virus-induced local inflammation elicits adaptive anti-viral and anti-tumoral immunity.

To test the hypothesis, we established a mouse glioma model in immunocompetent mice and intended to recapitulate adenoviral therapy in glioma patients. In our study, as it does in human glioma cells, Delta-24-RGD induces autophagy and cell lysis in mouse glioma cells (figure 1), providing the condition for the priming of immune cells to induce adaptive anti-glioma immunity in vivo [19], [20], [27]. A challenge using Delta-24-RGD in the mouse glioma model is that the human Ad5, from which Delta24-RGD is originated [8], is usually deficient to produce infectious viral progeny in mouse cells [36] (figure 1E). To partially compensate the deficiency of Delta-24-RGD replication in GL261 cells, we administrated three injections of the virus (3×10^8^ pfu/injection) into the tumor. This strategy results in recruitment of immune cells at the tumor site (figure 2 and 3), induction of anti-glioma immunity (figure 4A), shrinkage of the tumor and extended lifespan of the animals (figure 3A and 4B). As a comparison, in a hamster HaK renal tumor model, the therapeutic effect is achieved after six injections of oncolytic adenovirus at a higher dose (1×10^10^ pfu/injection) [37].

One of the hallmarks of cancer is evasion of immune destruction [38]. For example, cancer cells may paralyze infiltrating CTLs and NK cells, by secreting TGF-β or other immunosuppressive factors [38]. Reversing the immune suppression within the tumor probably is required to elicit efficacious anti-cancer immunity. Our data show the intratumoral injections of Delta-24-RGD break the immunosuppressive balance within the tumor and stir up the stimulators for a robust Th1 immune response as indicated by the increment of T-bet expressing cells, including NK cells, in the tumor (figure 2A and B) [28]. The tumor cells are no longer shielded from host immune system, and consequently, the proinflammatory Th1 cytokines, such as IFNγ, may recruit adaptive CD4+ and CD8+ lymphocytes to the tumor site (figure 2B and 3B) to instigate an anti-glioma immunity (figure 4A). In addition, TAAs are more efficiently presented to activated immune cells in the context of viral infection. In the virus-infected tumor cells, MHC I and immunoproteasome subunits are upregulated by IFNγ [17], resulting in the increased TAA (OVA) presentation to CD8+ T cells (B3Z cells) (figure 5B and C). This is part of the reason why the splenocytes from mice with tumor treated by Delta-24-RGD react stronger against the TAAs presented on GL261 cells than the splenocytes from mice with untreated tumor (figure 4A). Furthermore, during actual adenoviral injection in the tumor, infected tumor cells could also present TAAs to other types of immune cells, such as CD4+ T helper cells, to regulate the immunity toward an adaptive anti-tumor one. Moreover, the immune setting in the virus-treated tumor also favors activated dendritic cells and macrophages (figure 2C) to present more efficiently the TAAs from the cell debris resulted from viral oncolysis to T cells. Therefore, specific anti-tumor adaptive immunity is induced by the virus, co-existing with anti-virus adaptive immune response (figure 4A). Then, the anti-viral immunity should wind down when the virus is cleaned up, while the anti-tumoral immunity lasts until the tumor is eliminated by the immune system. In fact, both of the two immune activities toward virus-infected cancer cells should benefit the eradication of the tumor cells to achieve tumor regression and extended survival of the animals (figure 4 B and C).

## Conclusion

Our data here, for the first time, demonstrate that a glioma model in immune competent mice for adenoviral therapy is feasible for studying the therapeutic effect of oncolytic adenoviruses. Our results show the intratumoral injections of the virus induce Th1 innate and adaptive immune response, resulting in specific anti-tumor immunity, tumor shrinkage and prolonged animal survival. Thus, although key in inactivating viruses, the host immune system can also act as an ally against tumors, interacting with oncolytic viruses under the right conditions to generate useful and long-lasting antitumor immunity.

With an in vitro OVA modeling system, we offer the first and direct evidence that adenoviral infection and IFNγ resulted from in vivo viral infection increase the presentation of TAA to CD8+ T cells through the ‘classic’ endogenous pathway of MHC I presentation.

Our studies here provide a feasible animal model to study the therapeutic effect of oncolytic adenoviruses in immune competent context that partially recapitulate the scenario in a clinical setting in glioma patients. It provides us a platform to test new adenoviral therapy regimen in the future. The mechanistic study of TAA presentation opens the avenue to new strategies to modulate the immunity in the host to achieve better response in patients.
